# In Memoriam—Dr. J. G. Willis

**Published:** 1871-06

**Authors:** James Leslie, H. A. Smith


### IN MEMORIAM.

At a meeting of dentists, held at the office of Dr. Berry, the following resolutions were adopted, expressive of the sense of the profession in regard to the death of Dr. J. G. Willis:

*Resolved*. That in the very sudden and unexpected death of Dr. J. G. Willis, of this city, we regard a marked instance of the inscrutable working of His hand to whom we are all responsible and subject.

*Resolved*, That we can not but lament the loss of a member of our profession of such marked ability, both natural and acquired. Of him much was expected by his profession, and that expectation was always honored. He stood highest in the estimation of those who knew him best.

*Resolved*, That in his death we in common with all who knew him suffer the loss of a valued and useful member of society—one whose kind hearted geniality and Christian, gentlemanly bearing could not but be recognized by all.

*Resolved*, That we tender to the bereaved family our most sincere sympathy, and express to them the great sadness we can not but share with them.

JAMES LESLIE, *Chairman*.

H. A. SMITH, *Secretary*.

